# Identification of an 11-Autophagy-Related-Gene Signature as Promising Prognostic Biomarker for Bladder Cancer Patients

**DOI:** 10.3390/biology10050375

**Published:** 2021-04-27

**Authors:** Chaoting Zhou, Alex Heng Li, Shan Liu, Hong Sun

**Affiliations:** Department of Environmental Medicine, New York University School of Medicine, 341 East 25 Street, New York, NY 10010, USA; Chaoting.Zhou@nyulangone.org (C.Z.); Alex.Li@nyulangone.org (A.H.L.); shan.liu@nyulangone.org (S.L.)

**Keywords:** bladder cancer, autophagy, gene signature, overall survival, The Cancer Genome Atlas

## Abstract

**Simple Summary:**

Human bladder cancer, one of the most common cancers worldwide, is a molecularly heterogenous and complex disease. Identifying novel prognostic biomarkers and establishing new predictive signatures are important for personalized medicine and effective treatment of bladder cancer patients. Autophagy, a cell self-maintenance process that removes damaged organelles and misfolded proteins, displays both tumor promotion and suppression activities. The aim of our study is to investigate the function of autophagy-related genes in bladder cancer with the main focus on their contribution to prognostic outcome. By analyzing data obtained from The Cancer Genome Atlas (TCGA), we identified 32 autophagy-related genes that were highly associated with overall survival of bladder cancer patients. Further statistical assessment established an 11-autophagy-related-gene signature as an effective prognostic biomarker to predict the survival outcomes of bladder cancer patients.

**Abstract:**

Background: Survival rates for highly invasive bladder cancer (BC) patients have been very low, with a 5-year survival rate of 6%. Accurate prediction of tumor progression and survival is important for diagnosis and therapeutic decisions for BC patients. Our study aims to develop an autophagy-related-gene (ARG) signature that helps to predict the survival of BC patients. Methods: RNA-seq data of 403 BC patients were retrieved from The Cancer Genome Atlas Urothelial Bladder Carcinoma (TCGA-BLCA) database. Univariate Cox regression analysis was performed to identify overall survival (OS)-related ARGs. The Lasso Cox regression model was applied to establish an ARG signature in the TCGA training cohort (N = 203). The performance of the 11-gene ARG signature was further evaluated in a training cohort and an independent validation cohort (N = 200) using Kaplan-Meier OS curve analysis, receiver operating characteristic (ROC) analysis, as well as univariate and multivariate Cox regression analysis. Results: Our study identified an 11-gene ARG signature that is significantly associated with OS, including *APOL1*, *ATG4B*, *BAG1*, *CASP3*, *DRAM1*, *ITGA3*, *KLHL24*, *P4HB*, *PRKCD*, *ULK2*, and *WDR45*. The ARGs-derived high-risk bladder cancer patients exhibited significantly poor OS in both training and validation cohorts. The prognostic model showed good predictive efficacy, with the area under the ROC curve (AUCs) for 1-year, 3-year, and 5-year overall survival of 0.702 (0.695), 0.744 (0.640), and 0.794 (0.658) in the training and validation cohorts, respectively. A prognostic nomogram, which included the ARGs-derived risk factor, age and stage for eventual clinical translation, was established. Conclusion: We identified a novel ARG signature for risk-stratification and robust prediction of overall survival for BC patients.

## 1. Introduction

Bladder cancer (BC) is one of the most common cancers worldwide, with an estimated 550,000 new cases (almost 425,000 in males and over 125,000 in females) in 2018 [1]. More than 60% of all bladder cancer cases and half of all the 165,000 bladder cancer deaths occur in the less developed regions of the world. Bladder cancer is a molecularly heterogeneous and complex disease [2,3]. Approximately 30% of cases of invasive bladder cancer are associated with occult distant metastasis at the time of diagnosis, leading to a disappointing 5-year survival rate in patients with muscle-invasive bladder cancer [1]. Environmental and occupational exposure to risk factors such as cigarette smoking, chemical carcinogens, arsenic in drinking water, and endemic chronic urinary infections with *Schistosoma haematobium* are the main causes of bladder cancer [4]. Among these, cigarette smoking is the top risk factor, accounting for 50–65% of male cases and 20–30% of female cases [5,6]. The prognosis of patients with invasive bladder cancer is still very poor. The most common histopathological prognostic variables are tumor stage and lymph node density [7]. Recently, the Cancer Genome Atlas (TCGA) classified bladder cancer into five molecular subtypes (luminal-papillary, luminal-infiltrated, luminal, neuronal, and basal-squamous), each of which has distinct differentiation patterns, oncogenic mechanisms, tumor microenvironments, and clinical and histological associations [8]. The luminal-papillary, luminal-infiltrated, and luminal subtypes highly express luminal markers, including *GATA3, UPK1A, UPK2, KTT20, FGFR3*, etc. The extracellular matrix (ECM) and smooth muscle genes are highly expressed in the luminal-infiltrated subtype. True to name, the neural subtype highly expresses neural differentiation genes. Basal squamous subtypes highly express squamous epithelium-like genes (*GSDMC*, *TGM1*, etc.), immune marker genes (*L1CAM*, *CXCL11*, etc.), and basal marker genes (*CD44*, *KRT5*, *KRT6A*, etc.). The molecular diversity of subtypes contributes to the different responses to conventional and targeted therapies [9]. Thus, biomarker identification is of importance for diagnosis and prognosis of bladder cancer in the genomic era [10].

Autophagy is an evolutionarily conserved “self-eating” process, in which aberrant intracellular proteins and damaged organelles are engulfed in double-membrane structures and subsequently delivered to lysosomes for degradation. Both natural and pathological processes, including nutrient deprivation, organelle damage, and chemotherapy or radiation therapy of cancer cells, are able to trigger autophagy [11,12,13]. In cancer, autophagy functions as a double-edged sword with both tumor-promoting and tumor-suppressing activities, depending on tumor type, stage, and genetic context [14]. In early tumor formation, autophagy acts as a tumor suppressor by maintaining genomic integrity, preventing cell proliferation and inflammation. However, once established, tumor cells utilize autophagy to survive [15,16].

Very few studies have reported the function of autophagy in human bladder cancer. Dickstein et al. reported that AZ7328, an AKT inhibitor, induced autophagy in human bladder cancer cell lines, which protected cancer cells from AZ7328-induced apoptosis [17]. In addition, maternally expressed gene 3 (MEG3) has been reported to be downregulated in bladder cancer tissues compared to normal controls, resulting in increased cell proliferation and autophagy activity [18]. In contrast to the tumor promotion activity shown by these two studies, autophagy has been recently reported to be able to inhibit growth and invasion of bladder cancer cells [19,20]. Downregulation of high-mobility group AT-hook1 (HMGA1) and miR-211 in bladder cancer cells induced cytotoxic autophagy and suppressed cell proliferation, migration, and invasion [19]. Treating bladder cancer cells with isorhapontogenin, a small stilbene derivative, induced autophagy and inhibited cell growth and invasion via the MAPK8-JUN-SESN2 axis [20]. However, the molecular pathways that mediate autophagy’s tumor promotion or tumor suppression activities in bladder cancers are not fully characterized. Gene expression signatures for survival stratification in bladder cancer patients have been proposed by various studies [21,22,23,24]. A recent study has reported a 7-autophagy-gene signature in bladder cancer patients [25]. Four out of seven genes were significantly downregulated in bladder cancer compared to the control group, and displayed high sensitivity and specificity in bladder cancer diagnosis [25]. However, a prognosis-related autophagy-related gene (ARG) signature has not been reported in bladder cancer. Thus, for this paper, we aimed to investigate the function of autophagy-related genes in bladder cancer and their contribution to prognostic outcomes using a data-based approach. By analyzing RNA-seq data of bladder cancer patients and their clinical follow-up information, obtained from TCGA, we identified the prognosis-related ARGs that were highly associated with overall survival. Then, we used multiple statistical analysis methods to identify the potential correlation of these genes and prognostic outcome. We eventually built a convenient nomogram to predict survival rate by combining the risk score of autophagy-related genes and major confounding factors.

## 2. Methods

### 2.1. Data Acquisition

A total of 213 autophagy-related genes were obtained from the HADB database (Human Autophagy Database, http://autophagy.lu/clustering/index.html, accessed on 1 May 2021), which provides a comprehensive and up-to-date list of autophagy-related genes and proteins (Table S1). The RNA sequencing (RNA-seq) data and corresponding clinical follow-up information were extracted from the Bladder Carcinoma (TCGA-BLCA) database (https://cancergenome.nih.gov, accessed on 1 May 2021) database, a project that was initiated in 2005 by the National Cancer Institute to identify genetic mutations implicated in cancer using genome sequencing and bioinformatics. Out of 414 bladder cancer patients with the clinic follow-up data (ranging from 0 to 5050 days) collected in the TCGA Urothelial Bladder Carcinoma (TCGA-BLCA) database, our study included 403 BC patients with a minimum follow-up duration of more than one month. Univariate Cox regression analyses were performed to identify ARGs associated with overall survival of patients for subsequent model construction.

### 2.2. Functional Analysis

To investigate a comprehensive set of functional annotation of prognosis-related ARGs, gene ontology (GO) term enrichment analysis and Kyoto Encyclopedia of Genes and Genomes (KEGG) pathway analysis were performed using the “Clusterprofiler” package in R.

### 2.3. Construction of an ARGs-Related Prognostic Model

The prognostic value of autophagy-related genes of all samples was computed by a univariate Cox regression analysis. Then, samples were randomly divided into two cohorts—the training cohort (N = 203) and the validation cohort (N = 200) (Table S2). The least absolute shrinkage and selection operator (LASSO) method was used to identify gene signatures and obtain their respective coefficients value. After incorporating expression values for each particular gene, a risk score formula for each patient was constructed and weighted according to its estimated regression coefficients in a multivariate Cox regression analysis. The prognostic risk score for predicting overall survival was calculated as follows:

$$Risk\;score=\overset{n}{\underset{i=1}{\sum }}exprgene\left(i\right)\times Coeffgene\left(i\right)$$

According to the risk scoring formula, the median risk score in the training cohort and the validation cohort was used as the cutoff value. Bladder cancer patients in each cohort were divided into low-risk and high-risk groups. The overall survival curves of the two groups were generated through the “survival” package in R. 1-, 3-, and 5-year receiver operating characteristic (ROC) curve analyses were performed to estimate the predictive value of the candidate genes. Sensitivity and specificity of the risk model of CESC were calculated by the AUC of the ROC curve with “survival ROC” R package. A *p* value < 0.05 was considered statistically significant in the prognostic signature analysis.

### 2.4. Statistical Analysis

Overall survival differences in the low- and high-risk groups in each cohort were evaluated by the Kaplan–Meier curve and compared by log-rank statistical methods. The median values were used as cut-off thresholds to plot the KM curves, and the statistical significance was evaluated by the log rank test. Both multivariate Cox regression analysis and stratification analysis were implemented to examine the role of risk scores in predicting patient outcomes. The ‘glmnet’ R package (version 2.0–16) was employed to perform the least absolute shrinkage and selection. The univariate and multivariate analysis was enforced using the Cox proportional hazard regression model. A nomogram for predicting the OS was built using the R library “rms” package. The survival probabilities were predicted by receiver operating characteristic (ROC) curve analysis for both 3- and 5-year survival. The validation of the nomogram-based prediction model was accessed via bootstrapped calibration curves and quantified as a C-index. The “B” and “m” parameters were set as 200 and 30, respectively. All statistical analyses were performed using the R language (version 3.6). A *p*-value of < 0.05 was identified as statistically significant.

## 3. Result

### 3.1. Identification of Prognostic Autophagy-Related Genes

**Figure 1:**
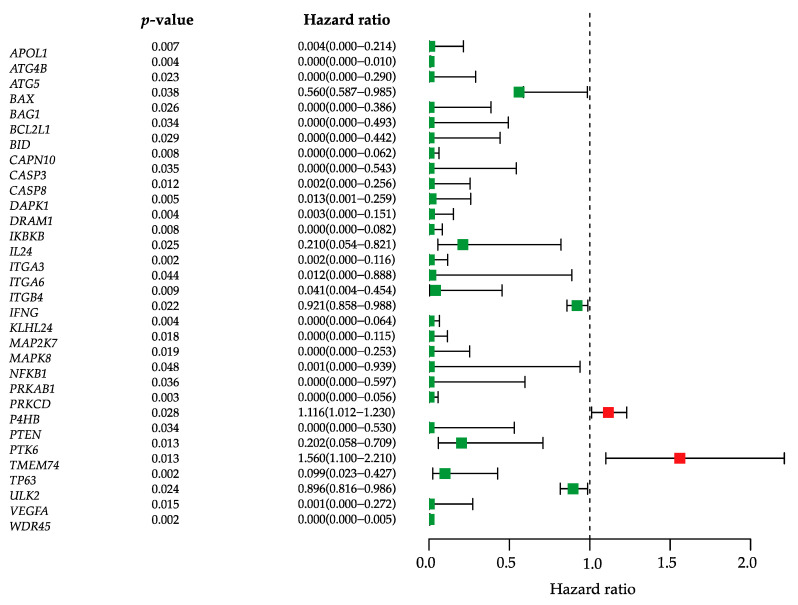
Identification of prognostic autophagy-related genes. Univariate Cox regression analysis obtained 32 ARGs. The hazard ratio forest plot shows the prognostic value of each gene. Green squares represent genes with hazard ratio <1, while red squares represent genes with hazard ratio >1. Horizontal lines are 95% CIs.

We obtained the 213 autophagy-related genes (ARGs) (Table S1) from the Human Autophagy Database (HADB, http://autophagy.lu/clustering/index.html, accessed on 1 May 2021) and merged them with RNA sequencing (RNA-seq) data of 403 BLCA samples on the Illumina HiSeq RNA-Seq platform from The Cancer Genome Atlas Urothelial Bladder Carcinoma (TCGA-BLCA) database. The demographic and clinical features of patients were listed in Table 1. Univariate Cox regression analysis was performed to identify prognostic ARG biomarkers, and yielded 32 genes that were significantly associated with the overall survival in bladder cancer (Figure 1). Most of these genes have hazard ratio below 1, which means that they are protective factors. The two genes with hazard ratio higher than 1 were P4HB and TMEM74. While P4HB has been recently identified as a novel prognostic biomarker associated with overall survival of bladder cancer patients [26], TMEM74 has been shown to promote tumor cell survival by inducing autophagy. High expression of TMEM74 significantly shortens the surviving periods of patients in several types of cancer [27].

### 3.2. Identification of Involved Signaling Pathways and Functional Annotation

**Figure 2:**
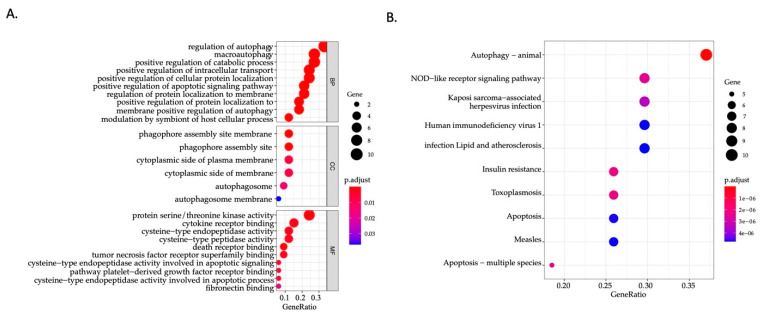
Functional annotation related to the 32 prognostic ARGs in bladder cancer. (**A**) GO analysis of 32 prognostic ARGs. (**B**) KEGG pathway enrichment of 32 prognostic ARGs. The size of the circle represents the number of genes. The color of the circle represents *p*-value. GO, Gene Ontology; KEGG, Kyoto Encyclopedia of Genes and Genomes; ARG, autophagy-related genes.

To investigate potential signaling pathways and potential function related to the 32 ARGs in bladder cancer (Table 2), we explored their biological characteristics and pathways using Gene Ontology (GO) enrichment analysis as well as the Kyoto Encyclopedia of Genes and Genomes (KEGG) pathway analysis. GO enrichment analysis of 32 prognostic ARGs provides a biological understanding of these genes. Biological function analysis (Figure 2A) showed that these genes were significantly associated with regulation of autophagy, macroautophagy, positive regulation of catabolic process, and positive regulation of apoptotic signaling pathway. According to cellular component enrichment, these genes were mainly located in the cytoplasmic side of plasma membrane, the phagophore assembly site membrane, and the autophagosome. Molecular function analysis suggested that the major functions of these genes were protein serine/threonine kinase activity, cytokine receptor binding, cysteine-type endopeptidase activity, cysteine-type peptidase activity, death receptor binding, and tumor necrosis factor receptor superfamily binding. KEGG analysis (Figure 2B) showed that pathways of prognostic autophagy-related genes were mostly enriched in autophagy, followed by the NOD-like receptor signaling pathway, the Kaposi sarcoma-associated herpesvirus infection, and the human immunodeficiency virus 1 infection.

### 3.3. Identification of Autophagy-Related Gene Signatures

**Figure 3:**
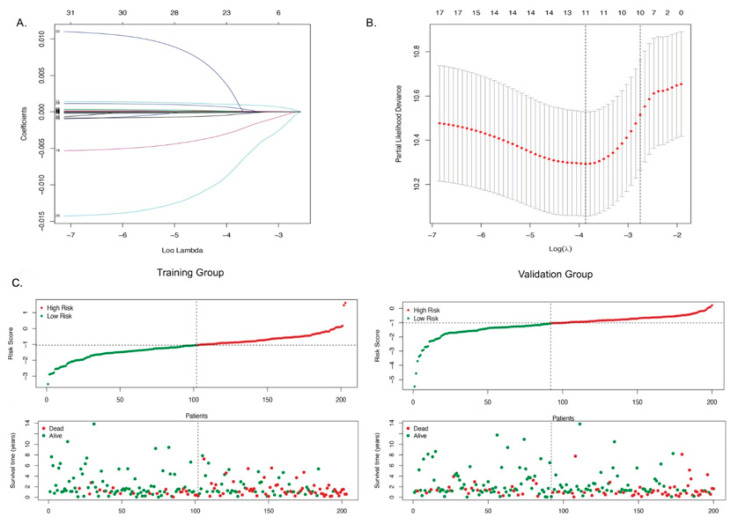
Expression profile and prognostic value of ARGs. (**A**) LASSO coefficient spectrum of 11 genes in BLCA. The coefficient distribution map was generated for a logarithmic (λ) sequence. (**B**) Selection of the best parameters for BLCA in the LASSO model (λ). (**C**) Distribution of prognostic index (upper panel) and survival status of patients (lower panel) in different groups.

It has been reported that autophagy plays a vital role in bladder cancer progression [28,29]. To test whether autophagy-related genes can be used as prognostic biomarkers, we applied the Least Absolute Shrinkage and Selection Operator (LASSO) Cox regression model to the 32 genes extracted from RNA-seq data of bladder cancer patients. We randomly divided the 403 patients into two cohorts, a training cohort (N = 203) and a validation cohort (N = 200) (Table S2). A LASSO regression model was then used to select key prognosis-associated genes. In the LASSO-penalized Cox regression, the corresponding coefficients of certain genes were reduced to zero along with log λ (a tuning parameter) changed, indicating that they were shrinking parameters and their effects on the model could be omitted (Figure 3A). Following cross-validation, 11 genes achieved the minimum partial likelihood deviance (Figure 3B). Moreover, at this point, log λ was approximately -3.9, and the 11 genes displayed non-zero effects. These genes are *APOL1*, *ATG4B*, *BAG1*, *CASP3*, *DRAM1*, *ITGA3*, *KLHL24*, *P4HB*, *PRKCD*, *ULK2,* and *WDR45*. Using the multivariate Cox regression analysis, we established an autophagy-clinical prognostic index (API) based on the 11-gene prognostic model. Then we calculated the risk score for each individual patient using API and the mRNA expression of 11 genes (Table S2). Patients with a risk score higher than the median were classified into the high-risk group and those with a risk score lower than the median were classified into the low-risk group. Figure 3C showed the distribution of risk scores in patients with bladder cancer and the correlation between survival time and risk score in training cohorts. Patients with higher score were correlated with higher number of dead cases, which indicated that high-risk patients generally had poor overall survival. Similar trending was identified in validation cohorts (Figure 3C). Further analysis revealed that a significant correlation exists between the mRNA expression of 8 genes (*APOL1*, *ITGA3*, *WDR45*, *CASP3*, *PRKCD*, *ATG4B*, *ULK2,* and *P4HB*) and overall survival of bladder cancer patients (Figure S1). In addition, 10 out of 11 genes (except *CASP3*) exhibited a significant difference in mRNA expression levels between high- and low-risk groups (Figure S2).

### 3.4. The 11-ARG Signature as an Independent Risk Factor for Bladder Cancer

**Figure 4:**
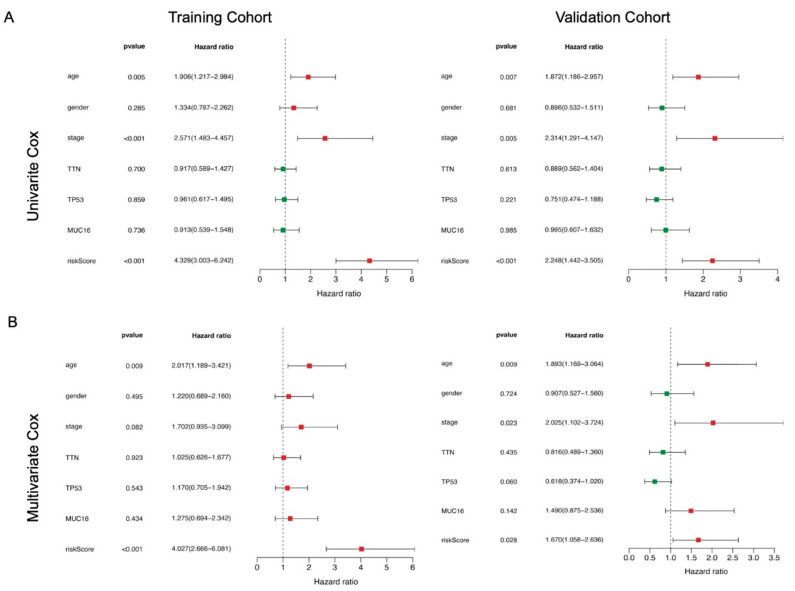
Prognostic indicators based on ARGs show good predictive performance and development of a prognostic index based on ARGs. A forest plot of (**A**) univariate and (**B**) multivariate Cox regression analysis in the training group and the validation group. Green squares represent genes with hazard ratio <1, while red squares represent genes with hazard ratio >1. Horizontal lines are 95% CIs.

**Figure 5:**
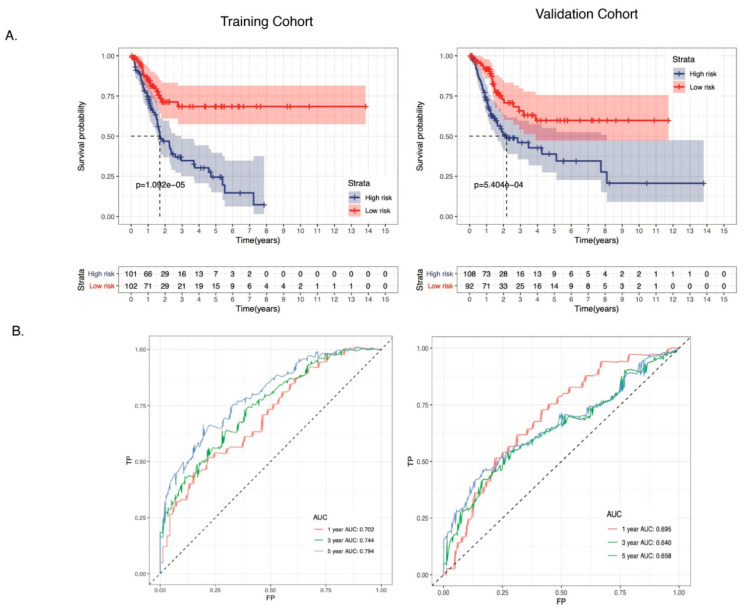
Evaluation of the 11-gene ARG signature in TCGA training and validation cohorts. (**A**) The Kaplan–Meier survival curve analysis revealed a shorter overall survival in the high-risk group. The blue line represents the high-risk group. The red line represents the low-risk group. (**B**) Survival-dependent ROC curves validated the prognostic significance of ARGs-based prognostic indicators. The red line represents the 1-year AUC; the green line represents the 3-year AUC; and the blue line represents the 5-year AUC. ROC, receiver operating characteristic; AUC, the area under the ROC curve.

We performed univariate and multivariate Cox regression analyses on this 11-gene panel by including various clinical variables (e.g., age, gender, stage) of bladder cancer patients, as well as the mutation status of *TNN*, *TP53*, and *MUC16*, which are the top 3 mutated genes in bladder cancer (Figure S3). Univariate Cox regression analysis showed that API was significantly associated with patient prognosis in both training cohorts (*p*-value <0.001) and validation cohorts (*p*-value < 0.001) (Table 3, Figure 4A). Clinicopathological features including age, gender, stage, as well as mutation status of *TTN*, *TP53,* and *MUC16* were further adjusted by multivariate Cox regression analysis. API remained an independent prognostic indicator for bladder cancer in training and validation cohorts with *p*-value < 0.01 and <0.05, respectively (Figure 4B). Kaplan–Meier overall survival curve analysis was plotted to determine the performance of the API in predicting clinical outcomes in bladder cancer patients. As shown in Figure 5A, the survival rate of patients in the high-risk group was significantly lower than that in the low-risk group in both training (*p*-value < 0.001) and validation (*p*-value < 0.001) cohorts (Figure 5A). Then, survival-dependent receiver operating characteristic (ROC) curves were performed to validate the accuracy of ARGs-based prognostic indicators. For the training group, the areas under the ROC curve (AUC) for 1-year, 3-year, and 5-year survival rates were 0.702, 0.744, and 0.794, respectively (Figure 5B). For the validation group, the AUC for 1-year, 3-year, and 5-year survival rates were 0.695, 0.640, and 0.658, respectively (Figure 5B). These results indicated that the ARGs-based prognostic indicators have a robust potential in survival prediction for bladder cancer patients.

### 3.5. Establishment of a Nomogram for Predicting Survival in Bladder Cancer Patients

**Figure 6:**
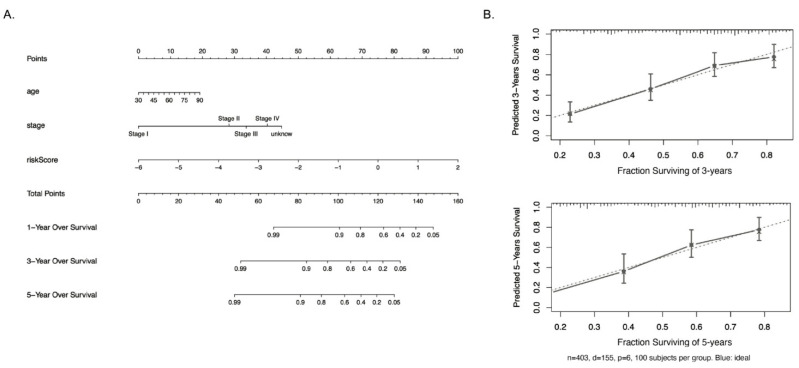
Nomograms predicting survival in bladder cancer patients. (**A**) The nomogram to predict 1-, 3-, and 5-year overall survival of bladder cancer patients was created based on three independent factors. (**B**) The 403-patient bootstrapped calibration plots show the comparison of predicted and actual overall survival probabilities at 3- and 5-year follow-up. The dotted line represents the ideal fit; circles represent nomogram-predicted probabilities; stars represent the bootstrap-corrected estimates; and error bars represent the 95% CIs of these estimates.

In order to further improve the prediction accuracy and practicability of API, we established a risk nomogram, which combined our 11-ARGs gene signature and other univariate significant features, for predicting the survival probability in bladder cancer patients. As shown in Figure 6A, a higher total score calculated by the sum of different factors in the nomogram was indicated with worse 1-year, 3-year, and 5-year overall survival rates. For example, a 60-year-old stage II BLCA patient with API of -2 would yield a total of 87.5 (10 points for age, 27.5 points for stage II, and 50 for -2 API score), with predicted 1-year, 3-year, and 5-year overall survival rates around 0.92, 0.80, and 0.73. Figure 6B showed calibration plots for the prediction of 3- and 5-year OS rates, which demonstrated a robust similarity between observed outcomes and predicted survival probabilities. These results strongly suggest our ARGs signature combined with age and bladder cancer stages have an overall superior clinical predictive power for determining survival outcomes in bladder cancer patients.

## 4. Discussion

As the prognosis of bladder cancer is still poor, new strategies are urgently needed to be established to achieve better prognosis performance and increase overall survival of bladder cancer patients. Biomarkers are reliable prognostic methods for identifying the oncologic outcome including disease progression in patient and overall survival. In this study, we identified the autophagy-related genes that were significantly correlated to overall survival of bladder cancer patients. Among these prognostic autophagy-related genes, we discovered a novel 11-gene ARG signature that could classify bladder cancer patients into two distinct groups, high-risk and low-risk groups, with different clinical and molecular features. The prognostic power of the identified ARG signature was further validated in both training and validation groups. Furthermore, we combined this with patient clinical information, including age and bladder cancer stage, to establish an easy-to-use risk-assessment nomogram, which can predict 1-year, 3-year, and 5-year overall survivals of bladder cancer patients.

Several studies have highlighted the biomarker signatures associated with prognosis for bladder cancer patients. Chen et al. identified a four-gene signature from the gene expression profiles of 93 bladder cancer patients from the GEO database and 408 bladder tumor patients from TCGA database [30]. Dancik and Theodorescu performed a gene set enrichment analysis of 1968 patients with lung, bladder, or head and neck cancer, and identified 10–15 cell cycle-related genes as a predictive signature [31]. Mo et al. reported an 18-gene signature to characterize two major tumor differentiation subgroups, “basal” and “differentiated,” based on gene expression profiles from three bladder tumor datasets (TCGA, MDA, and LUND) [32]. In the context of cancer, dysfunction of autophagy may lead to increased free radicals and aggregated macromolecules, which may result in elevated DNA damage, mutation, metabolic deficiencies, and global cellular damage. On the other hand, abnormal autophagy could help cancer cells resistant to cell death. For these reasons, autophagy-related genes could be promising predictors in cancer. In the present study, we identified 32 ARGs that were significantly associated with the overall survival of bladder cancer patients. Among these genes, 11 ARGs (*APOL1*, *ATG4B*, *BAG1*, *CASP3*, *DRAM1*, *ITGA3*, *KLHL24*, *P4HB*, *PRKCD*, *ULK2,* and *WDR45*) displayed a high potential in predicting clinical outcome of bladder cancer patients. When dividing the patients into high- and low- risk groups based on median risk score calculated from these 11 ARGs, the high-risk bladder cancer patients exhibited significantly poor overall survival, while the low-risk patients generally have better overall survival. In addition, to further improve the prognostic accuracy of the 11-ARGs signature, we combined it with clinical features including age and stage. This method is much more potent than single factor prognosis methods.

Among genes identified in 11-ARG gene signature, *ATG4B*, *WDR45,* and *DRAM1* play an important role in autophagy regulation. *ATG4B* is a core autophagy protein involved in formation of autophagosomes [33]. Recent studies suggest that *ATG4B* is a potential biomarker and vital in cancer therapy through the regulation of autophagy [33,34]. WDR45 has been shown to regulate autophagy via the AMPK and mTOR pathways and may also modulate autophagosome size [35]. *DRAM1* is a p53 target gene and a regulator of the ATG5-independent autophagy pathway [36,37]. It has been reported to induce migration and invasion abilities of glioblastoma stem cells [38]. *BAG1, PRKCD*, and *CASP3* are important regulators of apoptosis and recently were found to participate in autophagy. *BAG1*, a BCL2-associated athanogene, binds to oncogene *BCL2* and enhances its anti-apoptotic effects. It has been reported that *BAG1*, associated with LC3-II, may participate in the induction of autophagy through interaction with Hsc70 [39]. Dysregulation of BAG1 has been reported in many human cancers, including breast cancer, non-small cell lung cancer, glioblastoma, etc. [40]. Several studies on breast cancer suggested that *BAG1* is a prognostic marker in breast cancer [40,41]. Protein kinase C delta isoform (*PRKCD*) belongs to the protein kinase C (PKC) family, which consists of serine/threonine protein kinases and is a critical regulator of cell proliferation, survival, and cell death [42]. Recent studies have reported its potential role in modulating the crosstalk between apoptosis and autophagy. It has been reported that *PRKCD* activated GSK3αβ by phosphorylation and inhibited autophagy in cadmium-exposed NRK52E kidney cells [43]. Consistently, Rottlerin, an effective inhibitor of *PRKCD*, induced autophagy and caused apoptosis in breast cancer stem cells [44]. *PRKCD* has been shown to suppress breast cancer cell migration [45,46]. The potential role of *BAG1* and *PRKCD* in bladder cancer has not been reported. In contrast, *CASP3*, caspase 3, has been recognized as a prognostic predictor in bladder cancer [47,48,49]. As an important effector caspase, *CASP3* not only plays a crucial role in apoptosis [47] but also modulates autophagy through the cleavage of core autophagy proteins, such as Beclin-1, *ATG5*, and *ATG4D* [50,51,52]. *APOL1*, apolipoprotein L1, is a BH3-only protein and belongs to the APOL family of proteins [53]. APOLs are predominantly involved in the regulation of lipid transport and metabolism [54,55], and are expressed in papillary thyroid carcinomas [56]. *APOL1* has been reported to induce autophagy and autophagy-associated cell death in a variety of cell types [57]. *APOL1* genetic variants are reported to be a major drive of kidney diseases [58], but their function in bladder cancer has not been reported. *ITGA3* and *ULK2* have been studied in bladder cancer [59,60]. *ITGA3*, integrin alpha-3, was known to influence the development of various cancers including bladder cancer [60,61,62,63]. Upregulation of *ITGA3* was observed in clinical specimens and bladder cancer cell lines [60]. Silencing *ITGA3* inhibited tumor cell migration and invasion through regulating FAK/PI3K/AKT and FAK/Src signaling mechanisms [60]. *ULK2* is a serine/threonine kinase that participates in autophagosome formation and initiation of autophagy [64]. *ULK2* knockdown inhibited autophagy and apoptosis in bladder cancer cells [59]. ULK family proteins also have been used both as a direct or indirect target for tumor therapy [65,66,67]. *KLHL24* belongs to the Kelch-like gene family, which contains a bric-a-brac, tramtrack, broad complex (BTB)/poxvirus and zinc finger (POZ) domain that can function in transcriptional repression through interacting with Cullin3 to form E3 ligase and mediating the ubiquitination of substrate proteins [68]. *P4HB* is one of the hub genes in the beta subunit of prolyl 4-hydroxylase and play an important function in inhibiting the aggregation of misfolded proteins [69]. It has recently been reported as a novel diagnosis and prognosis marker for kidney renal clear cell carcinoma [70]. *P4HB* was found to be significantly correlated with a high frequency of *TP53* mutations in glioma cells [71]. It is worth noting that high *TP53* mutations is a hallmark of muscle-invasive bladder cancer [72]. Additionally, P4HB has been utilized as an autophagy-related prognostic marker for cancer [70,73,74].

When tested as individual gene, 9 genes from the 11-ARG signature (*APOL1*, *ATG4B*, *CASP3*, *ITGA3*, *P4HB*, *PRKCD*, *ULK2*, and *WDR45*) exhibited a significant association between mRNA expression levels and overall survival of bladder cancer patients (Figure S1). While higher expression of *APOL1*, *ATG4B*, *ITGA3*, *WDR45*, *CASP3,* and *PRKCD* is associated with favorable survival in human bladder cancer patients, increased *ULK2* and *P4HB* mRNA levels are negatively correlated to overall survival of bladder cancer patients. Despite the favorable survival rate observed in the high expression group of *DRAM1*, *BAG1*, and *KLHL24*, the correlations are less significant. Thus, our results suggest that survival analysis of individual genes might be insufficient in addressing the multilevel complexity in cancer patients. Consistent with the negative correlation to the overall survival of patients, the mRNA expression of *ULK2* and *P4HB* are significantly higher in the high-risk group than the low-risk group (Figure S2). The other 8 genes, except for *CASP3*, were significantly higher in the low-risk group compared to the high-risk group, which supports their correlation to favorable survival in bladder cancer patients. Indeed, cancer diagnosis and prognosis are challenging due to the complexity of gene expression alteration in individual patients. Identification of a multi-gene signature from cancer patients as well as a combination of the gene signature-derived risk factor and other clinical parameters will provide more robust evaluation of cancer prognosis.

Our study identified a novel 11-gene ARG signature that was significantly correlated with the overall survival of human bladder cancer patients. Based on the expression value of 11 ARGs, an autophagy-related prognostic index was generated and successfully divided the patients into low-risk or high-risk subgroups. Moreover, a nomogram model combining the risk score values and the tumor stage and age of the patients exhibited good predictive efficacy on the overall survival of bladder cancer patients. However, our study solely relies on the TCGA dataset by dividing the patients randomly to the testing and validation group. Due to this limitation, our model has not been validated in an independent dataset or in cohorts with different TMN stages. In addition, AUCs in our analysis are relatively low. Thus, the model’s clinical accuracy needs to be further assessed with more datasets and different sample attributes.

## 5. Conclusions

Taken together, our work provides a comprehensive, accurate, and convenient prognosis of the survival outcomes of bladder cancer patients. These genotypic risk assessment markers may serve to be useful in assessing patients with higher survival rates. This can assist in the creation of patient-specific treatment regimens that will improve patient care. At the same time, our results show great potential of innovative molecular targets for bladder cancer treatment strategies. For further studies, clinical application of this signature for the prognosis of bladder cancer patients are needed for further evaluation.

## Figures and Tables

**Table 1:** Characteristics of patients in TCGA database.

Variables	TCGA (N = 403)
**Status**	
Alive	249
Dead	154
Age	68 ± 10.57
**Gender**	
Female	102 (26.2)
Male	299 (73.8)
**Race**	
White	332 (82.5)
Asian	43 (10.6)
Black or African	28 (6.9)
**AJCC** **-T**	
T0/Ta	1 (0.2)
T1	3 (0.8)
T2	117 (28.9)
T3	193 (47.7)
T4	58 (14.3)
Unknown	31 (8.1)
**AJCC** **-N**	
N0	235 (58.0)
N1	46 (11.4)
N2	75 (18.5)
N3	7 (1.7)
Unknown	40 (10.4)
**AJCC** **-M**	
M0	195 (48.1)
M1	11 (2.7)
Mx	197 (49.2)
**Pathologic_stage**	
I&II	130 (32.1)
III&IV	271 (67.4)
Unknown	2 (0.5)
**Tumor_grade**	
G1/G2	20 (5.0)
G3/G4	380 (94.3)
Unknown	3 (0.7)

**Table 2:** Prognostic autophagy-related genes.

Id	HR	HR.95 L	HR.95H	*p*-Value
*APOL1*	3.68 × 10^−3^	6.33 × 10^−5^	2.14 × 10^−1^	6.87 × 10^−3^
*ATG4B*	5.23 × 10^−7^	2.63 × 10^−11^	1.04 × 10^−2^	4.18 × 10^−3^
*ATG5*	1.06 × 10^−4^	3.86 × 10^−8^	2.90 × 10^−1^	2.34 × 10^−2^
*BAX*	5.60 × 10^−1^	5.87 × 10^−1^	9.85 × 10^−1^	3.79 × 10^−2^
*BAG1*	3.33 × 10^−4^	2.86 × 10^−7^	3.86 × 10^−1^	2.61 × 10^−2^
*BCL2L1*	1.17 × 10^−4^	2.79 × 10^−8^	4.93 × 10^−1^	3.35 × 10^−2^
*BID*	3.72 × 10^−4^	3.14 × 10^−7^	4.42 × 10^−1^	2.88 × 10^−2^
*CAPN10*	2.97 × 10^−5^	1.42 × 10^−8^	6.20 × 10^−2^	7.52 × 10^−3^
*CASP3*	1.89 × 10^−4^	6.55 × 10^−8^	5.43 × 10^−1^	3.48 × 10^−2^
*CASP8*	1.99 × 10^−3^	1.55 × 10^−5^	2.56 × 10^−1^	1.21 × 10^−2^
*DAPK1*	1.27 × 10^−2^	6.27 × 10^−4^	2.59 × 10^−1^	4.52 × 10^−3^
*DRAM1*	2.98 × 10^−3^	5.88 × 10^−5^	1.51 × 10^−1^	3.70 × 10^−3^
*IKBKB*	6.90 × 10^−5^	5.82 × 10^−8^	8.18 × 10^−2^	7.97 × 10^−3^
*IL24*	2.10 × 10^−1^	5.38 × 10^−2^	8.21 × 10^−1^	2.48 × 10^−2^
*ITGA3*	2.35 × 10^−3^	4.75 × 10^−5^	1.16 × 10^−1^	2.34 × 10^−3^
*ITGA6*	1.16 × 10^−2^	1.51 × 10^−4^	8.88 × 10^−1^	4.40 × 10^−2^
*ITGB4*	4.12 × 10^−2^	3.74 × 10^−3^	4.54 × 10^−1^	9.19 × 10^−3^
*IFNG*	9.21 × 10^−1^	8.58 × 10^−1^	9.88 × 10^−1^	2.22 × 10^−2^
*KLHL24*	1.45 × 10^−4^	3.29 × 10^−7^	6.36 × 10^−2^	4.41 × 10^−3^
*MAP2K7*	2.72 × 10^−6^	6.43 × 10^−11^	1.15 × 10^−1^	1.84 × 10^−2^
*MAPK8*	1.91 × 10^−4^	1.44 × 10^−7^	2.53 × 10^−1^	1.95 × 10^−2^
*NFKB1*	8.46 × 10^−4^	7.61 × 10^−7^	9.39 × 10^−1^	4.80 × 10^−2^
*PRKAB1*	3.01 × 10^−4^	1.52 × 10^−7^	5.97 × 10^−1^	3.63 × 10^−2^
*PRKCD*	1.65 × 10^−4^	4.86 × 10^−7^	5.63 × 10^−2^	3.42 × 10^−3^
*P4HB*	1.12 × 10^−1^	1.01 × 10^−1^	1.23 × 10^−1^	2.80 × 10^−2^
*PTEN*	2.27 × 10^−4^	9.75 × 10^−8^	5.30 × 10^−1^	3.40 × 10^−2^
*PTK6*	2.02 × 10^−1^	5.76 × 10^−2^	7.09 × 10^−1^	1.26 × 10^−2^
*TMEM74*	1.56 × 10^−1^	1.10 × 10^−1^	2.21 × 10^−1^	1.32 × 10^−2^
*TP63*	9.94 × 10^−2^	2.31 × 10^−2^	4.27 × 10^−1^	1.91 × 10^−3^
*ULK2*	8.96 × 10^−1^	8.16 × 10^−1^	9.86 × 10^−1^	2.39 × 10^−2^
*VEGFA*	1.15 × 10^−3^	4.88 × 10^−6^	2.72 × 10^−1^	1.52 × 10^−2^
*WDR45*	6.04 × 10^−7^	7.23 × 10^−11^	5.04 × 10^−3^	1.88 × 10^−3^

*p <* 0.05 was considered statistically significant. HR, hazard ratio.

**Table 3:** Univariate and multivariate Cox regression analysis of clinical pathologic features for OS.

Variables	Univariate Cox Regression				Multivariate Cox Regression			
	Training Group		Validation Group		Training Group		Validation Group	
	HR	*p*-Value	HR	*p*-Value	HR	*p*-Value	HR	*p*-Value
Age	1.90599	0.00480	1.87244	0.00712	2.01716	0.00923	1.89282	0.00942
Gender	1.33404	0.28459	0.89624	0.68090	1.21995	0.49531	0.90684	0.72394
Stage	2.57129	0.00077	2.31364	0.00483	1.70227	0.08188	2.02544	0.02310
*TTN*	0.91655	0.69983	0.88862	0.61304	1.02461	0.92293	0.81568	0.43476
*TP53*	0.96076	0.85924	0.75083	0.22132	1.17030	0.54285	0.61782	0.04981
*MUC16*	0.91318	0.73605	0.99522	0.98484	1.27489	0.43391	1.48957	0.14201
Risk Score	4.32931	0.00000	2.24823	0.00035	4.02659	0.00000	1.67008	0.02760

*p <* 0.05 was considered statistically significant. HR, hazard ratio. Age (>65 vs. 65<). Gender (female vs. male). Stage (stage I&II vs. stage III&IV). *TNN, TP53, MUC16* (mutant vs. wildtype).

## Data Availability

The data presented in this study are available on request from the corresponding author.

## Supplementary Materials

The following is available online at: https://www.mdpi.com/article/10.3390/biology10050375/s1, Figure S1: The Kaplan–Meier survival curve analysis of individual genes of 11-gene ARG signature, Figure S2: Boxplot showing the mRNA expression levels of 11 genes in high-risk and low-risk groups, Figure S3: Summary of mutations in bladder cancer patients, Table S1: 213 autophagy-related genes (ARGs) obtained from the Human Autophagy Database, Table S2: Summary of patients’ survival information, 11-ARG signature genes expression and risk score in training and validation cohorts.
