# Supplementary material for: Identification of an 11-Autophagy-Related-Gene Signature as Promising Prognostic Biomarker for Bladder Cancer Patients
Source: Biology (Basel). 2021 Apr 27;10(5):375. doi: 10.3390/biology10050375 (PMC8146553; doi:10.3390/biology10050375)
Supplement: Supplementary file 1 [file biology-10-00375-s001.zip › Supplementary Figures.pdf]

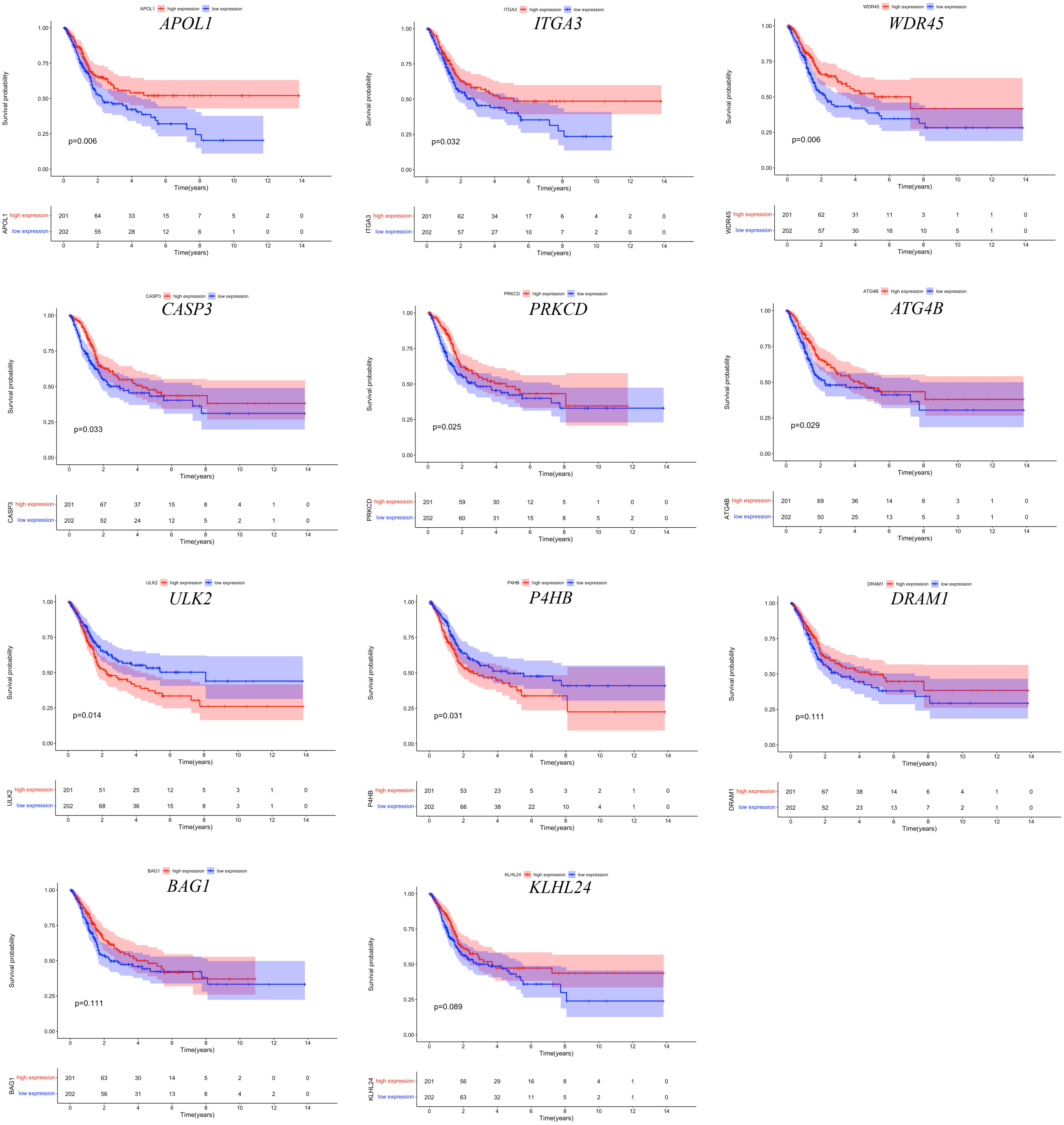

Figure S1. The Kaplan-Meier survival curve analysis of individual genes of 11-gene ARG signature, including *APOL1, ITGA3, WDR45, CASP3, PRKCD, ATG4B, ULK2, P4HB, DRAM1,BAG1,* and *KLHL24*. Group Cutoff= Median.

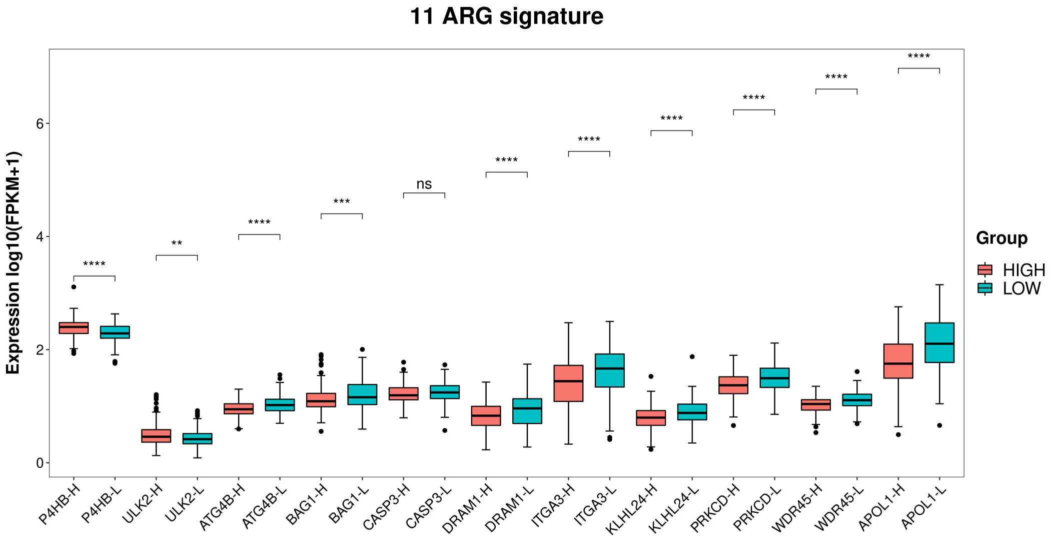

Figure S2. Boxplot show the mRNA expression levels of 11 genes (*P4HB, ULK2, ATG4B, BAG1, CASP3, DRAM1, ITGA3, KLHL24, PRKCD, WDR45* and *APOL1*) in high-risk and low-risk groups. ** $p \le 0.01$; *** $p \le 0.001$; **** $p \le 0.0001$; ns: not significant.

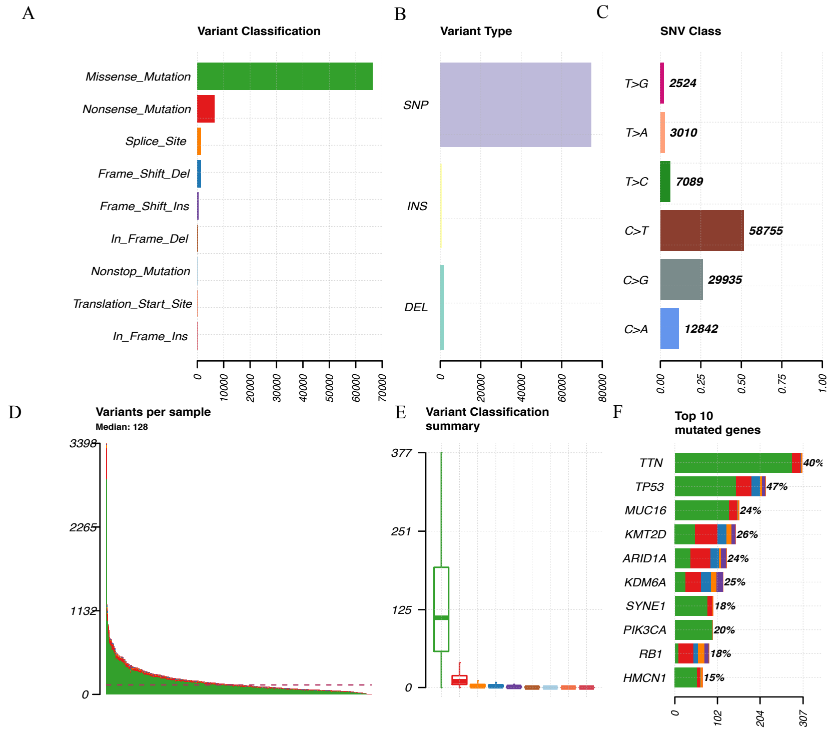

Figure S3. Summary of mutations in bladder cancer patient. Different variants in each sample were evaluated. The finding suggests that missense mutation (A), SNP (B), C>T (C) were more frequent. The number of variants per sample and variant classification were shown in (D) and (E), respectively. *TTN, TP53* and *MUC16* are the most frequently mutated genes (F).
